# Multipurpose contrast enhancement on epiphyseal plates and ossification centers for bone age assessment

**DOI:** 10.1186/1475-925X-12-27

**Published:** 2013-04-08

**Authors:** Hum Yan Chai, Tan Tian Swee, Gan Hong Seng, Lai Khin Wee

**Affiliations:** 1 IJN-UTM Cardiovascular Engineering Centre, Biotechnology Research Alliance, Faculty of Biosciences and Medical Engineering, Universiti Teknologi Malaysia, 81310 UTM Skudai, Johor Bahru, Johor, Malaysia; 2 Medical Implant Technology Group, Material and Manufacturing Research Alliance, Department of Biomechanics and Biomedical Materials, Faculty of Biosciences and Medical Engineering, Universiti Teknologi Malaysia, 81310, UTM Skudai, Johor Bahru, Johor, Malaysia; 3 Biomedical Engineering Group, Institute of Biomedical Engineering and Informatics, Faculty of Computer Science and Automation, Technische Univeristat Ilmenau, Ilmenau, Germany; 4 Department of Biomedical Engineering, Faculty of Engineering, University of Malaya, Kuala Lumpur, Malaysia

**Keywords:** Image contrast enhancement, Bone age assessment image quality metrics, Histogram partition, Brightness and detail preserving

## Abstract

**Background:**

The high variations of background luminance, low contrast and excessively enhanced contrast of hand bone radiograph often impede the bone age assessment rating system in evaluating the degree of epiphyseal plates and ossification centers development. The Global Histogram equalization (GHE) has been the most frequently adopted image contrast enhancement technique but the performance is not satisfying. A brightness and detail preserving histogram equalization method with good contrast enhancement effect has been a goal of much recent research in histogram equalization. Nevertheless, producing a well-balanced histogram equalized radiograph in terms of its brightness preservation, detail preservation and contrast enhancement is deemed to be a daunting task.

**Method:**

In this paper, we propose a novel framework of histogram equalization with the aim of taking several desirable properties into account, namely the Multipurpose Beta Optimized Bi-Histogram Equalization (MBOBHE). This method performs the histogram optimization separately in both sub-histograms after the segmentation of histogram using an optimized separating point determined based on the regularization function constituted by three components. The result is then assessed by the qualitative and quantitative analysis to evaluate the essential aspects of histogram equalized image using a total of 160 hand radiographs that are implemented in testing and analyses which are acquired from hand bone online database.

**Result:**

From the qualitative analysis, we found that basic bi-histogram equalizations are not capable of displaying the small features in image due to incorrect selection of separating point by focusing on only certain metric without considering the contrast enhancement and detail preservation. From the quantitative analysis, we found that MBOBHE correlates well with human visual perception, and this improvement shortens the evaluation time taken by inspector in assessing the bone age.

**Conclusions:**

The proposed MBOBHE outperforms other existing methods regarding comprehensive performance of histogram equalization. All the features which are pertinent to bone age assessment are more protruding relative to other methods; this has shorten the required evaluation time in manual bone age assessment using TW method. While the accuracy remains unaffected or slightly better than using unprocessed original image. The holistic properties in terms of brightness preservation, detail preservation and contrast enhancement are simultaneous taken into consideration and thus the visual effect is contributive to manual inspection.

## Introduction

Bone age assessment (BAA) is a clinical procedure commonly adopted by pediatrics radiologist to gauge the skeletal development in children and adolescents. Attributable to the infeasibility in examining our biological maturity by chronological age, the skeletal maturity or skeletal age invariably plays a pivotal role as an indicator for early detection of growth disorders. The future body height of children can as well be predicted using the skeletal age [1]. Among all the skeletal bones, the left hand X-ray radiograph is claimed [2] to be the effective skeletal maturity estimation. Therefore, it is traditionally selected to represent the biological maturity by analyzing features such as developments of ossification area and calcium position in the ossification area. Diseases of children related to endocrine disorders such as Hypothyroidism Cushing syndrome and constitutional growth retardation; chromosomal disorders such as Trisomy 18 and Turner syndrome; malnutrition such as Skeletal dysplasia and bone mineralization anomalies [3], can be early detected via analyzing the discrepancy between the skeletal age and biological age.

Currently, there are two major bone age assessments are being practiced [4]: Greulich-Pyle method (GP) [4] and Tanner-Whitehouse atlas (TW3) [5]. In the GP method, pediatricians inspects the hand bone radiograph and compares it with the radiographs in the atlas; bone age of hand bone radiograph in atlas that most resembles the patient's hand bone radiograph is assumed as the assessed bone age. Differently, the more recent TW3 method is a point collection index system. In this system, each ossification site is ranked by the level of development using several stages. Next, a specific score is assigned in accordance to the gender and the ossification site. Then, all the scores are totaled up. Finally, the total score is used to deduce the specific bone age via a conversion table.

The reliability and efficiency of the abovementioned approaches remain skeptical [6] as the procedures involve manual visual inspection and thus they are always associated with drawbacks such as being time-consuming and subjective [7,8]. Therefore, in recent years, numerous computer-aided BAA systems have been developed especially for TW2 and TW3 method that are more inherently suitable to be computerized [9]. However, the computerized system is not yet fully developed [10] due to the inherent problems in hand radiograph processing especially the low contrast in ossification area and high diversity in pixel intensity distribution. The contrasts of bone texture in ossification sites are of critical importance in the TW3 bone age assessment. An optimum contrast-adjusted ossification site facilitates subsequent procedures in computerized BAA system especially in segmentation. This is because if the outline of the bone structure is noticeable, and both the structure and texture of ossification bones are well-defined, then the pertinent features are visually and computationally distinguishable. Hence, a good contrast as preprocessing in computerized BAA system is prominent [11]. The research hypothesis, therefore, is to explore the question: can a contrast enhancement technique capable of enhancing the visual quality of the ossification sites comprehensively so that the required evaluation time in manual bone age assessment using TW method can be shorten. The scope of this research is limited in advancing the bi-histogram-based contrast enhancement technique on gray scale image by selecting a decomposition point that is capable of producing resultant image with holistic visual quality.

Histogram equalization (HE) is one of the most frequently implemented contrast enhancement techniques [12] due to its simplicity, low computational complexity, and effectiveness. The basic operation is performed by remapping the gray levels of the input image through a transform function created from cumulative density function. This results in a flattened and stretched dynamic range in resultant image histogram. The histogram equalized image normally contains fewer gray levels than input image. The interval gray levels between high histogram components are expanded and hence the overall contrast of the image is enhanced. In the next section, we review and evaluate the pros and cons of the existing histogram techniques and provide the motivation of our proposed algorithm.

### The review of existing histogram equalization

HE has been widely applied in various areas such as medical image processing [13], radar image processing and speech recognition. Despite its ability in image contrast enhancement, the conventional HE or Global Histogram Equalization (GHE) is not commonly applied [14] attributable to its low performance consistency as it tends to produce artifacts that deteriorate the visual quality of ossification bones. The gray scale stretching induces significant change of mean brightness in output image. Besides, the domination of high components of histogram over low components of histogram during HE leads to detail loss. Furthermore, [15] HE tends to produce over-enhanced output image if the gray level probability density encounters abrupt increments; this results in an unnatural visual effect and the loss of pertinent features in the ossification site. The undesirable visualization effect [16] has become the main challenge that impedes HE to be employed extensively.

To overcome the drawbacks, Mean Preserving Bi-histogram equalization (BBHE) [13] has been proposed. The HE method first segments the input image into two sub-images by its mean: The first part consists of the minimum gray level to mean; the second part consists of gray level from mean to the maximum gray level. Conventional HE is then performed independently on each sub-image. A similar HE method, namely Dualistic Sub-Image Histogram Equalization (DSIHE) [17] has been proposed. DSIHE segments the histogram based on the median, rather than using the mean value of image in BBHE. DSIHE is claimed to outperform BBHE in terms of brightness and entropy preservation. Next, Chen and Ramli [18] has proposed the MMBEBHE (Minimum Mean Brightness Error Bi-Histogram Equalization) — an optimal extension of BBHE. The MMBEBHE performs similarly to BBHE except that MMBEBHE evaluates the brightness preserving ability of the BBHE adopting each gray level as separating point. The gray level that capable of producing the least average mean brightness error (AMBE) is then eventually employed to perform the BBHE.

Recursive Mean Separate Histogram Equalization (RMSHE) [19] has been proposed. This is actually the iterative version of BBHE. RMSHE first separates the histogram using image mean; the process is then repeated in the sub-histogram; conventional HE is then performed independently on each sub-histogram similar to DSIHE and BBHE. The number of repetition depends on the parameter, r, where r is any positive integer that is specified by the user prior to the execution. The parameter r determines the number of sub-histogram. The technique produces 2^*r*^ sub-histograms and thus when the r is one, the RMSHE is exactly the BBHE. Thus RMSHE can be perceived as a generalized representation of BBHE. A similar recursive HE method, namely recursive sub-image histogram equalization (RSIHE) [20], has been proposed. The only difference to RMSHE is that the RSIHE divides the histogram according to the median. It, thus, could be viewed as an iterative extension of DSIHE. Both the recursive methods demonstrate that the brightness of image can be better preserved than the previous non-recursive HE method. However, there are a few limitations of these methods: the methods can only segment the histogram into sub-histograms in the power of two; as the number of sub-histogram increases, the contrast enhancement becomes increasingly insignificant, and therefore, despite better brightness preservation, it fails to achieve the ultimate goal of HE. Furthermore, the number of the sub-histogram requires manual user manipulation.

Similar drawbacks persist in the Recursively Separated and Weighted Histogram Equalization (RSWHE) [21]. The method decomposes histogram into multiple sub-histograms based on either mean or median as separating point, denoted as RSWHE-M and RSWHE-D respectively. Firstly, the method performs the histogram segmentation, then the histogram weighting module modifies the histograms by implementing the normalized power law function. Eventually the conventional HE is executed on each modified sub-histogram. The method is claimed to have achieved the brightness preservation and contrast enhancement.

Various improvements, extensions and generalizations of HE can be found in substantial papers. Apparently, most of the abovementioned literatures attempt to preserve the brightness and detail while performing the contrast enhancement by histogram equalization. However, the aforementioned methods are more inclined in addressing merely one of them and neglect the other. For instance, the methods BBHE, MMBEBHE, RMSHE, and RSIHE focus on the brightness preservation with less consideration on its detail preservation. Similarly, the clipping methods of [22] and [23] design the algorithms based on detail preservation without emphasizing on the problem of brightness deviation critically. Neither of them is practically applicable on the ground that brightness preservation methods tend to lose its information and detail (over-enhancement) whereas detail preserving methods are likely to alter the brightness of an input image and thereby lead to undesirable artifacts. To surmount the mentioned drawbacks, [24] and [14] endeavor to address the both brightness and detail preservation problems. The result, nevertheless, is not optimized in a broader sense.

Researchers attempted to preserve the brightness by decomposing the histogram of input image. Two decisions, however, arise when decomposition is performed: The Separating point and the number of sub-histograms. It can be easily proved [13] that the output mean of conventional histogram equalization does not consider the mean brightness of input image — the fundamental motivations that the histogram should be segmented to bind the equalized sub-images around the input mean. The motivation of [17] to segment the histogram using median is based on the information theory: entropy is at its maximum when the two sub-images have equal area. Mathematically [19], the histogram equalized output image mean converges to input image mean brightness. Therefore methods in [19-21] undergo recursive separation before histogram equalization is taken place in each sub-histogram. The purpose [25,26] of segmenting the histogram using local maximum and local minimum is to avoid the manual determination of recursive partition number.

This paper aims to present an optimal solution to enhance the contrast, the brightness preservation and detail preservation of output image simultaneously. A new histogram equalization method is proposed, named Multipurpose Beta Optimal Bi-histogram Equalization (MBOBHE). This paper disagrees with traditional improvements of histogram equalization that prone to optimize by minimizing or maximizing either one of the properties such as contrast, brightness preservation and detail preservation. Instead, we proposed histogram equalization that is capable of considering all the properties simultaneously by using a multiple-criteria objective function. The motivation for this design is that a visually good contrast image should have overall performance that takes all properties into account in order to produce more natural and comprehensive output image.

### Multipurpose optimized Beta bi-histogram equalization

The main steps in the proposed MBOBHE are summarized in 6 steps: 1) Decomposes an input image into two sub-images using each possible separating point. 2) Subsequently, perform histogram equalization independently in each sub-histogram. 3) Define three metrics to gauge mean brightness preservation, enhanced contrast and details retention. 4) Form the objective function by combining three defined components using weighted-sum approach. 5) Compute the function output by iterating each possible decomposition point for bi-histogram equalization. 6) Eventually, select resultant image of which the objective function is maximized.

### The construction of objective function

The gist of the proposed histogram equalization lies in the design of an objective function to regularize different metrics. To form the objective function, the relations of each property to the ideal expected result has to be verified. The ideal histogram equalized resultant hand bone radiograph is such that the image mean brightness difference between input and output image should be as close as possible, while it is still capable of assuring the significance of contrast enhancement to retain the pertinent information of the ossification sites in the hand bone image. Apparently, it is not a trivial task to govern automatically the enhancement intensity to fulfill the mentioned multiple properties simultaneously. Therefore, we claim our contribution in accomplishing this task.

In this section, we explain the intuition of our designed regularization function by first introducing briefly the conventional measurement of histogram equalized image quality. Then, we explain the unsuitability of those conventional performance metrics as component in multiple objectives function in assessing the comprehensive quality of histogram equalized image. After that, we propose better performance metrics as the components of multiple objectives function to manipulate the extent of operation performed by histogram equalization. Finally, we derive the regularization function by combining all the design components to consider multiple properties simultaneously.

### The components of objective function and Beta distribution function

On the ground of the abovementioned drawbacks of conventional performance metrics, we designed a new framework of quality metrics that are capable of quantitatively modeling the brightness preservation, contrast and detail change after the histogram equalization operation. We termed the three metrics as Brightness Preservation Score (BPS) function, Optimum Contrast Score (OCS) function, and Detail Preservation Score (DPS) function. These functions are modeled via Beta functions. Beta distribution works well in specifying various relationships between random variables to model expert opinions by having various function shapes over a certain desired range. Note that the modeling functions are not limited by beta function, any arbitrary functions that are capable of exhibiting desired relations can be implemented. However, well-known distribution functions are preferred for the sake of potential utility since the statistical properties of the function have been rigorously explored and comprehended. In fact, many distributions are instances of beta distribution, therefore, we suggested, without the loss of generality and flexibility, to select beta distribution in realizing the proposed algorithm. Each metric is then combined together to form the complete model of our objective function which then iterates over all possible gray level to search the separating grey level that maximizes the objective function.

### The Brightness Preservation Score (BPS) function

We designed the BPS as a numerical score characteristic function output that models the ability of brightness preservation corresponding to the brightness difference between the input image and the histogram equalized image. The function was designed such that the range of relative brightness difference (RBD) forming the function domain and the resultant values of BPS are bound within the range of 0 and 1. The step-by-step construction details of BPS function are discussed as below:

First we construct the brightness mean of input image, *μ*_*x*_ and mean of output image, *μ*_*y*_, defined mathematically as follow:

(1) $$\mu_{x}=\frac{1}{\mathrm{MN}}\sum_{i=0}^{M}\sum_{j=0}^{N}I_{x}\left(i,j\right)$$

(2) $$\mu_{y}=\frac{1}{\mathrm{MN}}\sum_{i=0}^{M}\sum_{j=0}^{N}I_{y}\left(i,j\right)$$

(3) $$\mathrm{RBD}=e^{\frac{\left|\mu_{y}-\mu_{x}\right|}{\mu_{x}+c}}$$

Where M and N denote the image dimensions. *I*_*x*_(*i*, *j*) denotes input image pixel’s intensity at spatial location of (*i*, *j*) and *I*_*y*_(*i*, *j*) denotes output image pixel’s intensity at spatial location of (*i*, *j*). Both *μ*_*x*_ of (1) and *μ*_*y*_ of (2) are required to construct function (3) which denotes the relative brightness difference (RBD) between the input radiograph and histogram equalized radiograph. The constant ‘c’ in the denominator represents a small constant that prevent the computational errors in extreme cases when *μ*_*x*_ = 0. It is obvious that the output value can be as large as infinity in extreme cases when the brightness mean of input radiograph is 0. *BPS*(*RBD* : *m*, *n*) denotes the remapping of RBD using beta distribution function with the parameters m and n.

(4) $$\begin{array}{c} \mathrm{BPS}\left(\mathrm{RBD}:m,n\right)=\frac{Г\left(m+n\right)}{Г\left(m\right)Г\left(n\right)}\mathrm{RB}D^{\left(m-1\right)}{\left(1-\mathrm{RBD}\right)}^{\left(n-1\right)}\\ \mathrm{Where}\;Г\left(x\right)=\overset{1}{\underset{0}{\int }}e^{-t}\mathrm{RB}D^{\left(x-1\right)}d\left(\mathrm{RBD}\right) \end{array}$$

(5) $$\mathrm{NBPS}=\frac{\mathrm{BPS}}{\mathrm{argmax}\left(\mathrm{BPS}\right)}$$

Equation (4) denotes the Brightness Preserving Score (BPS) function in the context of histogram equalization which bears a resemblance to Beta probability distribution function where RBD satisfies *x* ∈ [0, 1];. The establish fair comparison among components, BPS is normalized to map a RBD onto the unit interval [0,1]. We termed this normalized BPS as NBPS, defined in equation (5) as the ratio of BPS and maximum value of BPS over the range of RBD:

What is RBD? RBD is one way of quantifying the mean brightness difference between input image and output image. Then why is RBD not used directly as component to gauge brightness difference in objective function but BPS or the NBPS? The main concept is that small mean brightness difference, as pursued by previous researcher, does not assure a resultant image that gives favors human visual perception as a whole. Therefore, we need to remodel the brightness difference into a new characteristic function, the BPS or the NBPS.

What NBPS indicates? NBPS is a normalized function that maps RBD to a new set of values bound between 0 and 1. This mapping is based on beta distribution function with parameters m and n. Now the new paradigm becomes : ‘the nearest the resultant image contain NBPS that approaches value of 1, the more likely the resultant image conform to human visual perception’; instead of the traditional paradigm: ‘the smallest the value of brightness difference, the better it is the resultant image’. In short, The NBPS provides us an index to measure quantitatively the ability of brightness preservation of the histogram equalized algorithm. Other way of saying it, NBPS function regularizes the definition of 'good' or 'bad' brightness preserving ability, in terms of RBD.

Why NBPS important in improving the visual effect of ossification site and how it influent the observer visual perception? One important feature of the proposed NBPS is that it has revolutionized the traditional perception for brightness preservation. Traditionally, better brightness preserving ability associates with minimizing the brightness difference. We consider this perception is incorrect in the context of bone age assessment on the ground that small brightness difference might indicate negligible contrast enhancement and this in turn obscures the critical features in evaluating and assigning score for ossification centers and epiphyseal plate development. NBPS redefined the value of brightness preservation to produce resultant image without artifacts that impede the manual inspection.

### The Optimum Contrast Score (OCS) function

Last section describes how we quantify mean brightness difference and how we give merit to each score of RBD by mapping it to a new function of NBPS. However, mean brightness difference only constitutes one of the components of the final objective function. Multiple components are necessary to predict a more comprehensive perception by considering various factors that contribute to the final perception.

Similar to BPS in gauging mean brightness difference, the Optimum Contrast Score (OCS) is designed as a numerical score function that gauges the contrast enhancement corresponds to the contrast change between the input image and the histogram equalized image. This function is designed such that the contrast change represents the function domain, p and q represent the parameters, and the bound values between 0 and 1 represent the range. The detail of the function is discussed as follows:

First we construct the brightness mean of input image, *μ*_*x*_ and mean of output image, *μ*_*y*_, defined mathematically as follow:

(6) $$\sigma_{x}=\sqrt{\frac{1}{\mathrm{MN}}\sum_{i=0}^{M-1}\sum_{j=0}^{N-1}{(I_{x}\left(i,j\right)-\mu_{x})}^{2}}$$

(7) $$\sigma_{y}=\sqrt{\frac{1}{\mathrm{MN}}\sum_{i=0}^{M-1}\sum_{j=0}^{N-1}{(I_{y}\left(i,j\right)-\mu_{y})}^{2}}$$

Then, we defined the input of OCS or the relative contrast different (RCD) as follows:

(8) $$\mathrm{RCD}=1-e^{\frac{{}^{\left|\sigma_{y}-\sigma_{x}\right|}}{{}^{\sigma_{x}+c}}}$$

Where *σ*_*x*_ and *σ*_*y*_ denote normalized Root Mean Squared (NRMS) contrast of input image and output image, respectively, where pixel intensities are the i-th j-th element of the two dimensional image of size by M by N denoted by (6) and (7). C denotes an extremely small constant to maintain the function stability while not altering of function outcome significantly. We suggested the RCD function represents a contrast comparison metric. This function output satisfies bound condition where the range is limited so that *RCD* ∈ [0 1]; As a result, function output is 1 only when both input and output images possess identical standard deviation; function output is 0 whenever the NRMS of input image and output image are identical; the smaller the function output RCD, the larger the absolute difference (in terms of NRMS) between input and output image, and vice versa.

Then the definition of OCS and NOCS, as well as the relation between RCD and NOCS, are analogous to RBD and NBPS in last section; *OCS*(*RCD* : *p*, *q*) denotes beta-distribution-function modeled RCD with parameters p and q.

(9) $$\begin{array}{c} \mathrm{OCS}\left(\mathrm{RCD}:p,q\right)=\frac{Г\left(p+n\right)}{Г\left(p\right)Г\left(q\right)}\mathrm{RC}D^{\left(p-1\right)}{\left(1-\mathrm{RCD}\right)}^{\left(q-1\right)}\\ \mathrm{Where}\;Г\left(x\right)=\overset{1}{\underset{0}{\int }}e^{-t}\mathrm{RC}D^{\left(x-1\right)}d\left(\mathrm{RCD}\right) \end{array}$$

(10) $$\mathrm{NOCS}=\frac{\mathrm{OCS}}{\mathrm{argmax}\left(\mathrm{OCS}\right)}$$

This is to satisfy the new paradigm that distinguishable ossification centers and epiphyseal plates should possess a certain range values of contrast (note: henceforth, we termed these values as optimum values, which refer to values range that give highest scores of function; as mentioned, this location of this range depends on parameters of function) that opposes the traditional perception such that the value of contrast should be as high as possible. The property of triangular shaped distribution with correctly tuned parameters satisfies this new perception.

The new concept bases on the observation that relatively low contrast or relatively high contrast is inferior, but only certain ‘optimum’ values contrast is favorable. The hypothesis is that relatively low contrast might obscure the pertinent features in ossification centers and epiphyseal plates of which the extracted information are significant in determining the degree of developments; contrarily, relatively high contrast produce over-enhanced contrast artifacts on the ossification sites; Only ‘optimum’ contrast is able to emphasize and highlight the pertinent information so that it is remarkable in the ossification sites for efficient manual inspection by GP method.

### The Detail Preservation Score (DPS) function

We have defined two components of the objective function. This section, we define the last component --The Detail Preservation Score (DPS) -- that output a numerical score to gauge the detail preservation ability of histogram equalization corresponds to the simultaneous discrepancy of pixel-mean intensity between the input image and the histogram equalized image. The DPS function is designed such that the simultaneous pixel-mean intensity change (we termed it as average structural different (ASD)) represents the DPS function domain. The detail of the DPS function is discussed as follows:

Firstly, we define the input of DPS which is the ASD as follows

(11) $$d\left(x_{i,j},y_{i,j}\right)=\left(x_{i,j}-\mu_{x}\right)\left(y_{i,j}-\mu_{y}\right)$$

(12) $$\mathrm{ASD}\left(X,Y\right)=1-\frac{1}{\mathrm{MN}}\sum_{i=0}^{N}s\left(x_{i,j},y_{i,j}\right)\left\{\begin{array}{c} 1\;\mathrm{if}\;d\left(x_{i,j},y_{i,j}\right)\geq 0\\ 0\;\mathrm{if}\;d\left(x_{i,j},y_{i,j}\right)<0 \end{array}\right)$$

*d*(*x*_*i*,*j*_, *y*_*i*,*j*_) denotes the pixel-mean-difference function for input image, ***X*** and output image, ***Y***. Where *x*_*i*,*j*_ and *y*_*i*,*j*_ denote the normalized pixel's intensity of ***X*** and ***Y*** on (*i*, *j*) spatial location, respectively, of two dimensional image of size by M by N; The ASD satisfies bound condition where the range is bound between 0 and 1; ASD function output achieves 1 only when all the pixels between ***X*** and ***Y*** possess identical pixel-mean relationship; function output achieves 0 if all the pixel-mean relationships between ***X*** and ***Y*** are completely in different sign. The structural change of ***X*** and ***Y*** are quantified by ASD by assuming that the particular pixel's intensity that less than or more than mean intensity of input image must remain the relationship in ***Y*.** This describes the structure of the image; we termed this structural change as detail loss.

The traditional logic was that the closer the value of ASD approaches 1, the better the detail preservation of the image; on contrary, the nearer the value in ASD approaches 0, the more inferior the detail preservation of the image. However, we suggest that this logic can be further improved by introducing the ‘optimum’ value, since if there is no structural change, the enhanced contrast might not be significant enough to distinguish features in the ossification sites. Therefore, analogous to BPS and OCS, ASD is converted to DPS and NDPS using beta distribution function with parameters u and v as followings, where *DPS*(*ASD* : *u*, *v*) denotes beta-distribution-function modeled ASD with parameters u and v.

(13) $$\begin{array}{l} \begin{array}{c} \mathrm{DPS}\left(\mathrm{ASD}:u,v\right)=\frac{Г\left(u+v\right)}{Г\left(u\right)Г\left(v\right)}\mathrm{AS}D^{\left(u-1\right)}{\left(1-\mathrm{ASD}\right)}^{\left(v-1\right)}\\ \mathrm{Where}\;Г\left(x\right)=\overset{1}{\underset{0}{\int }}e^{-t}\mathrm{AS}D^{\left(x-1\right)}d\left(\mathrm{ASD}\right) \end{array} \end{array}$$

(14) $$\mathrm{NDPS}=\frac{\mathrm{DPS}\left(\mathrm{ASD}:u,v\right)}{\mathrm{argmax}\left(\mathrm{DPS}\left(\mathrm{ASD}:u,v\right)\right)}$$

This is to satisfy our hypothesis that distinguishable ossification centers and epiphyseal plates should have optimum detail preservation ability which is oppose to the traditional perception that the value of detail preservation should be as high as possible. The property of triangular shaped distribution with correctly tuned parameters satisfies this new perception. The new perception is based on the observation that relatively low and high detail loss is inferior, only the optimum detail loss is favorable. The reason is that relatively high detail preservation implies low contrast enhancement in the pertinent features in ossification centers and epiphyseal plates. Contrarily, relatively low detail preservation produce artifacts on the ossification sites that distort the relative relation among pixels and diminish important features in ossification sites; only optimum detail preservation is able to retain the pertinent information during the process of histogram equalization.

### The manipulation of parameters in NBPS, NOCS and NDPS

Correctly tuned parameters are essential to provide correct relation between the measurements in RBD, RCD and ASD to the corresponding scores in NBPS, NOCS and NDPS respectively. The parameters setting of MBOBHE depends heavily on the applications. In the context of computerized bone age assessment, the parameters are set to maximize the visibility of the amount of pertinent information based on the verbal criteria as well as the line drawings in [5] using mean opinion score (MOS) [27] by radiologist and pediatrician.

To form the complete regularization function, the relations of each function to the ideal expected result need to be verified. The ideal scenario — the values of image mean brightness for input and output image is as close as possible to peak value of NBPS, while contrast enhancement is not of insignificance and should be as close as possible to the peak value of BPS, with condition that the image are not over-enhanced and the features of the objects in the input image are not diminished or distorted according to the NDPS.

Each criterion acts as constraint to restrict the remaining criteria. To find an optimal solution that can satisfy all objectives, a mathematical model incorporating all the criteria has to be established. The ideal case is to find the separating point, *x*_*s*_ that optimizes (optimizing in this context is minimizing) all the above mentioned three criteria but this is not possible in most of the cases. Therefore, the 'optimal' solution here refers to finding the *x*_*s*_ that can best satisfy these criteria. The final objective considering three criteria is formulated as below:

(15) $$\mathrm{NObj}\left(\mathrm{NBPS},\mathrm{NOCS},\mathrm{NDPS}\right)=\frac{\alpha \left(\mathrm{NBPS}\right)+\beta \left(\mathrm{NOCS}\right)+\phi \left(\mathrm{NDPS}\right)}{\left(\alpha +\beta +\phi \right)}$$

**Figure 1:**
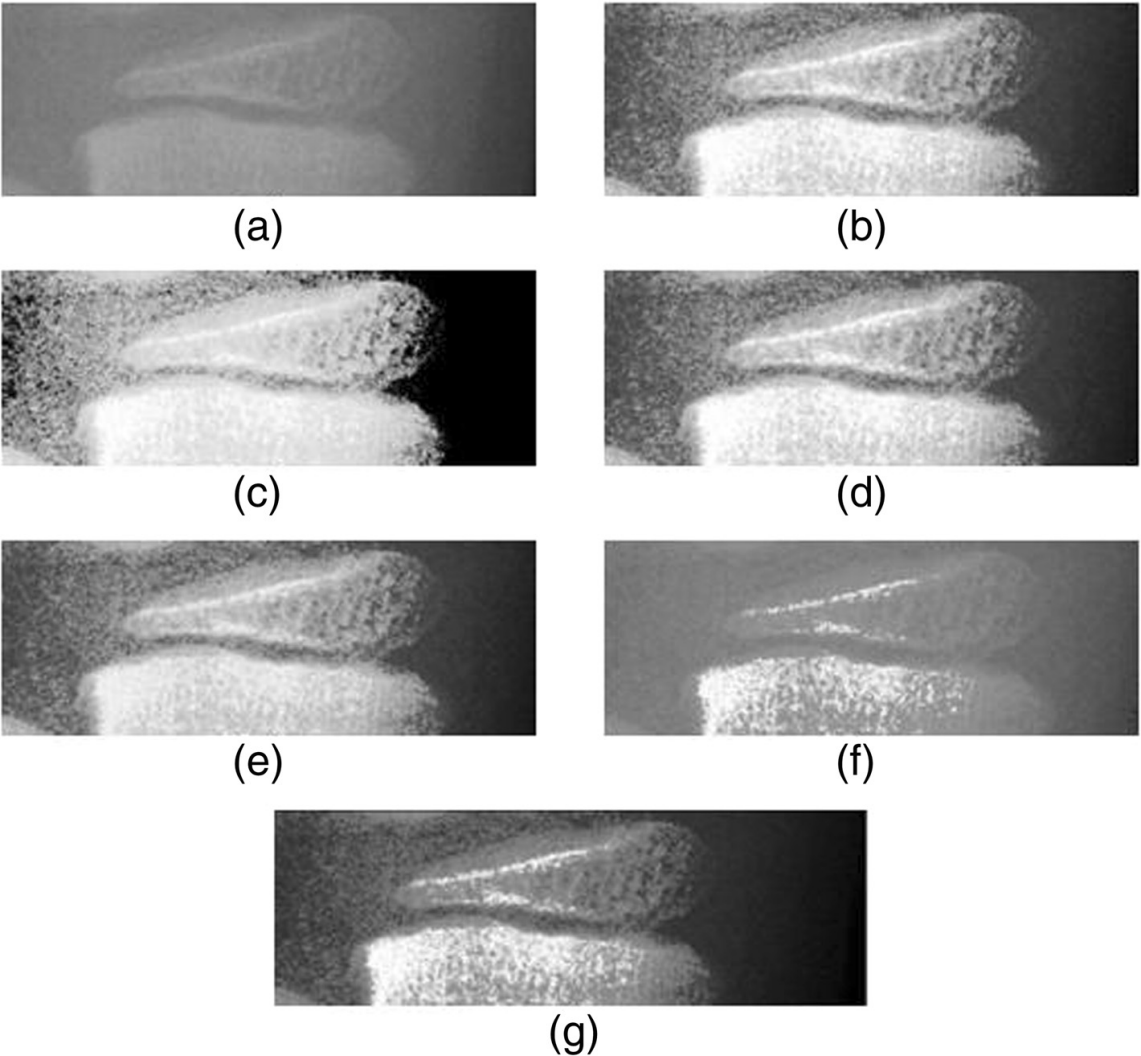
**Histogram equalization enhancement result for the radius ossification site using (a) Original Image (b) GHE (c) BBHE (d) DSIHE (e) MMBEBHE (f) RMSHE(r = 3) (g) MBOBHE.** This figure shows the enhancement result by showing the resultant image of an arbitrarily chosen site of ossification sites, which is radius, to illustrate the mentioned artifacts and effect of comprehensiveness produced by the proposed algorithm.

We evaluated the result of MBOBHE in the context of BAA using TW3 method via qualitative analysis by benchmarking the MBOBHE to existing types of HE methods: GHE, BBHE, DSIHE, MMBEBHE and RMSHE. All the recursive HE methods would perform using r = 3.The images used are ossification sites of hand bone of four ethnics comprising all ages from 0 to 17. The qualitative analysis is on case study basis by comparing the visual effect of resultant images by abovementioned methods. We explained the effect of artifacts of other conventional methods and the superiority of MBOBHE in contrast enhancement using arbitrary example in radius ossification site in Figure 1.

### Result demonstration

The original radiograph in Figure 1(a) is a particular stage of radius development where the epiphysis's proximal border has differentiated into palmar and dorsal surfaces with thickened white line at the proximal edge of the epiphysis. However, the contrast of original image is relatively low and hence the mentioned pertinent information used in bone age deduction is insufficiently distinguishable, neither to computerized BAA processing system nor to human inspector. Hence, this ossification site requires contrast enhancement to give prominence to the thickened white line in distal and proximal border of the epiphysis.

Figure 1(b), Figure 1(c), Figure 1(d) and Figure 1(e) show the resultant histogram equalized of the original image using the conventional GHE, BBHE, DSIHE, MMBEBHE respectively; the results illustrate the mentioned over-enhanced contrast artifacts on the ossification site that possibly involves extra computational expenses to computerized bone age assessment. This over-enhancement obscures human visual inspection on the information. Figure 1(f) shows the resultant histogram equalized image using RMSHE (r = 3); the result illustrates the detail loss artifacts in the medially and proximally grown thickened white lines; this diminished information is essential in determining the maturity stage of the radius ossification site. For the resultant histogram equalized image of MBOBHE in Figure 1(g), both thickened white lines adjacent to distal border and proximal border of epiphysis as well as the border edge of distal metaphysis are strengthened. The artifacts such as over-enhanced pertinent detail loss, however, are observed.

**Figure 2:**
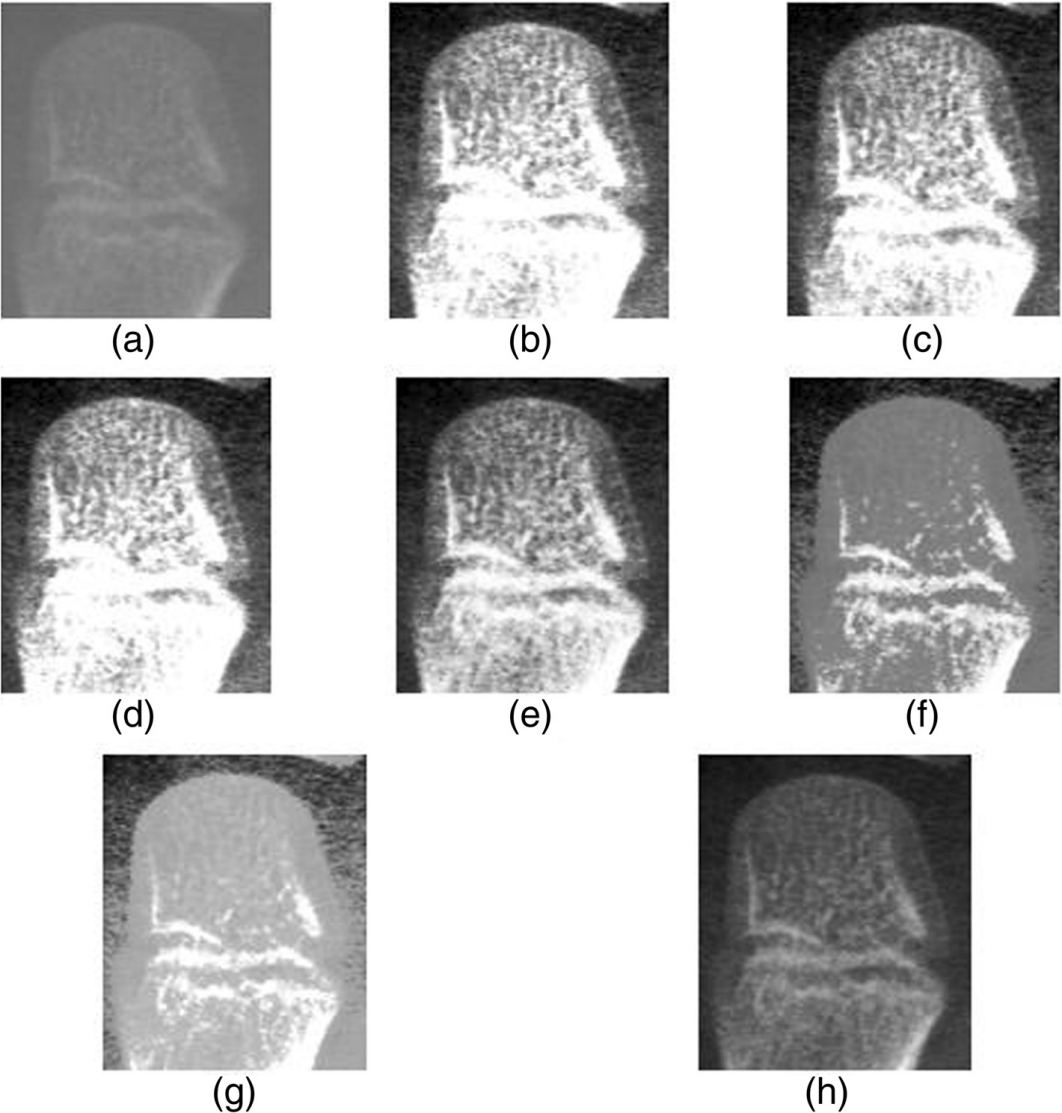
**Histogram equalization enhancement result for the third(III) of Metacarpals ossification site using (a) Original Image (b) GHE (c) BBHE (d) DSIHE (e) MMBEBHE (f) RMSHE(r = 3) (g) RSIHE(r = 3) (h) MBOBHE.** This figure shows the enhancement result by showing the resultant image of an arbitrarily chosen third (III) of Metacarpals ossification site to illustrate the mentioned artifacts and effect of comprehensiveness produced by the proposed algorithm.

The original radiograph in Figure 2(a) is in a particular stage of third (III) metacarpals development. The first feature is that the epiphysis has developed from semicircle into the shape of a spade with white edges in the lateral, medial and proximal borders of the dorsal surface in epiphysis. The second feature is that the longitudinal thickened white edges within the epiphysis as the palmar edges. However, the contrast of original image is relatively low and hence the mentioned pertinent information for deducing the bone age is insufficiently distinguishable by the angles observed at their junction. This affects the computerized BAA system to wrongly assign development stage and this indirectly affects the region-of-interest localization or segmentation process. Hence, contrast enhancement is necessary in this ossification site to highlight the medial, lateral and longitudinal thickened white line in distal and proximal border of the third (III) metacarpals epiphysis.

Figure 2(b), Figure 2(c) and Figure 2(d) show the resultant histogram equalized of the original image using the conventional GHE, BBHE and DSIHE respectively; the results illustrate the over-enhanced contrast artifacts on account of low brightness preservation ability on the ossification site which obliterates the important hints or information in development stage decision and become an interference in computerized BAA system. Resultant image using MMBEBHE in Figure 2(e) exhibits no severe over-enhancement but some insignificant texture in epiphysis has been strengthened which is not desirable. Figure 2(f) shows the resultant histogram equalized image using RMSHE (r = 3); the result illustrates the detail loss artifacts where edges of the epiphysis anatomical structure and the most of the textural structure within the epiphysis have been hindered.

Figure 2(g) demonstrates that the resultant histogram equalized image by RSIHE(r = 3) encounters the detail loss artifacts in lateral edges of the dorsal surface of the epiphysis, and unnecessary contrast enhancement in background bears the risk of obstructing both segmentation and region-of-interest localization in computerized BAA. For the resultant histogram equalized image of MBOBHE, all pertinent features can be inspected easily and no severe artifacts are observed after a moderate histogram equalization that has been regularized by multipurpose objective function.

**Figure 3:**
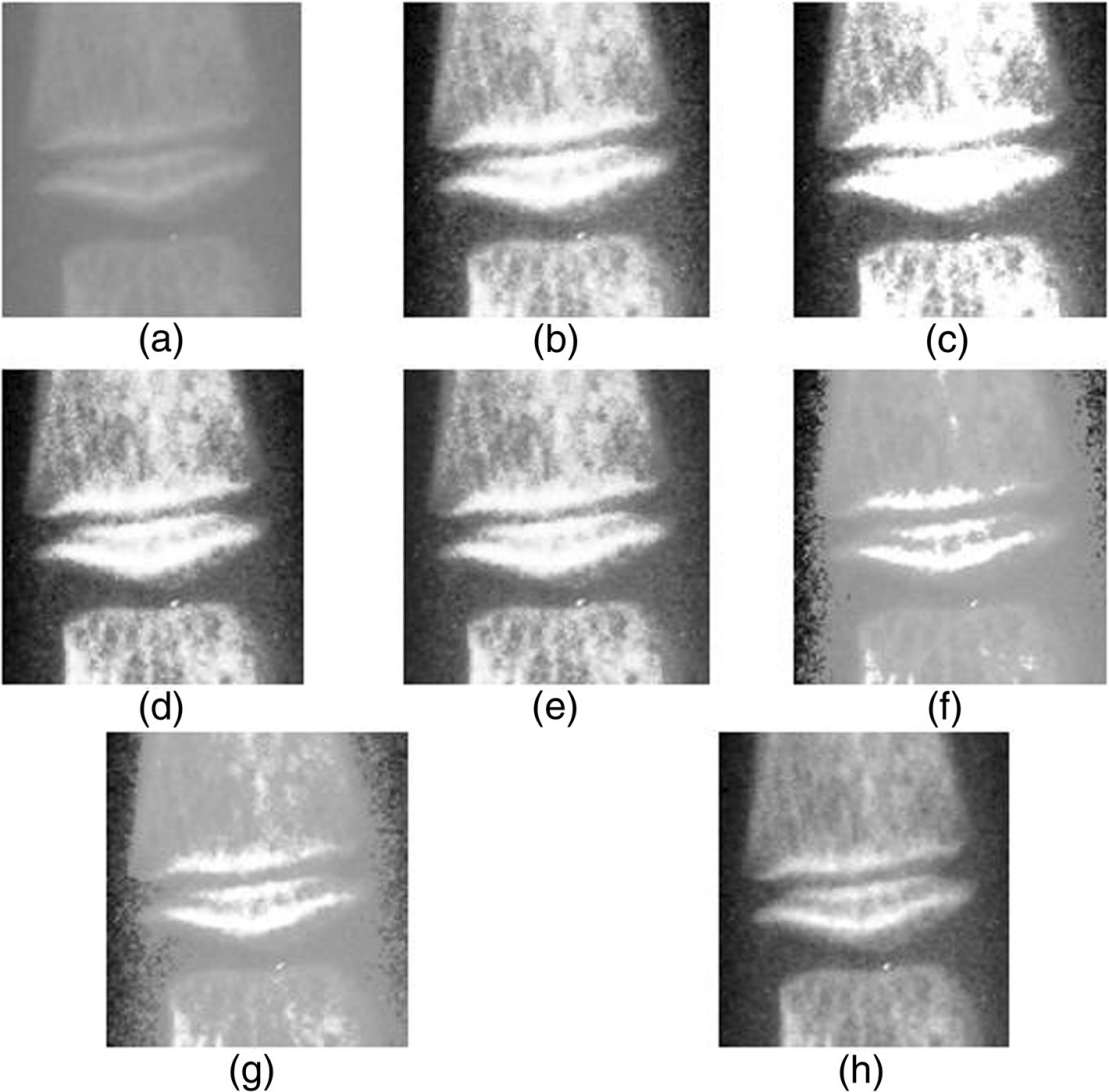
**Histogram equalization enhancement result for the third(III) of Middle Phalanx ossification site using (a) Original Image (b) GHE (c) BBHE (d) DSIHE (e) MMBEBHE (f) RMSHE(r = 3) (g) RSIHE(r = 3) (h) MBOBHE.** This figure shows the enhancement result by showing the resultant image of an arbitrarily chosen third (III) of Middle Phalanx ossification site to illustrate the mentioned artifacts and effect of comprehensiveness produced by the proposed algorithm.

The original radiograph in Figure 3(a) is a particular stage of third (III) Middle Phalanx development where the epiphysis is as wide as the metaphysis and the central portion of the Middle Phalanx's epiphysis proximal border has thickened and extended towards the end of the nearby phalanx forming the trochlear surface. White lines can be seen as a result of this thickening process. The white lines represent the dorsal surface and the palmar surface of the epiphysis. Nonetheless, insufficient contrast in the original radiograph leads to indistinguishable of the abovementioned pertinent information that is required in deducing bone age. This flaw from raw image without adequate contrast leads to false ossification site searching in computerized BAA and difficulty in manual inspection. Therefore, the original radiograph ossification site entails contrast enhancement to protrude the important features in stages determination.

Figure 3(b), Figure 3(c), Figure 3(d) and Figure 3(e) show the resultant histogram equalized of the original image using the conventional GHE, BBHE, DSIHE and MMBEBHE respectively; the results illustrate the over-enhanced contrast artifacts occasioned by failure in preserving the brightness of original image on the ossification site which hinders the pertinent information in deciding the stages and become an obstruct in computerized BAA system. Histogram equalized image using MMBEBHE in Figure 3(f) and Figure 3(g) exhibit no severe over-enhancement but the entire anatomical structure of Middle Phalanx are blended with background where the structures' border is indistinguishable. The shape of the epiphysis is unable to be recognized and analyzed and this leads to uncertainties in deciding the development stage where the ratio of the epiphysis and metaphysis is the determination factor. Figure 3(h) shows the MBOBHE processed resultant radiograph; the contrast between the bone and background has been greatly improved without severe problem of over-enhancement and detail loss; the pertinent features are salient enough for human inspector to analyze effortlessly and this increases the efficiency and reliability of computerized BAA.

The radiographs have been randomly selected as the qualitative analysis image to demonstrate the capability of MBOBHE due to its narrow dynamic range that could examine HE methods ability in preventing over-enhancement, detail and brightness preservation. The resultant image for each HE methods used in qualitative analysis has been shown. In general, it is observable that the original image has insignificant contrast enhancement and the important details are not sufficiently elicit. We observed that non-recursive methods, GHE, BBHE, DSIHE and MMBEBHE possess the inclination to exhibit over-enhancement in the contrast. For recursive methods, RMSHE(r = 3), it is noticeable that the RMSHE(r = 3) resultant image produces less washed-out effect but encounters details loss and distortion; For the proposed MBOBHE, the resultant image seldom exhibits over-enhancement effect, the detail of the epiphysis are always clearly observable. Furthermore, the brightness of the image bears relatively most resemblance to the input image compared to other methods in the qualitative analysis despite the contrast enhancement.

From the qualitative analysis, we found that GHE, BBHE, DSIHE are unable to display the ‘small’ features in image. This is mainly due to incorrect selection of separating point and having no extra processing techniques such as dynamic gray level allocation[28], histogram clipping [29] using plateau limit and histogram weighting [21]. For MMBEBHE, the separating point is selected by minimizing the AMBE. Therefore, the resultant image tends to perform the BBHE at the separating point that capable of giving the smallest luminance difference to the image without considering the contrast enhancement and detail preservation. This results in poor visibility of the features and hence leads to inaccuracies in computerized BAA and manual human inspection.

### Summary

Note that the measurement on each image property is not limited by RBD, RCD or ASD; it can be any arbitrary metric that is capable of gauging the property such that AMBE or entropy: Note also that these metrics can be further generalized by adding extra parameters or altering the parameters. However, both mentioned issues rely heavily on application, input data and user preference; in-depth discussion on which are beyond the scope of this article for the sake of having more focus on the general idea of this article.

**Figure 4:**
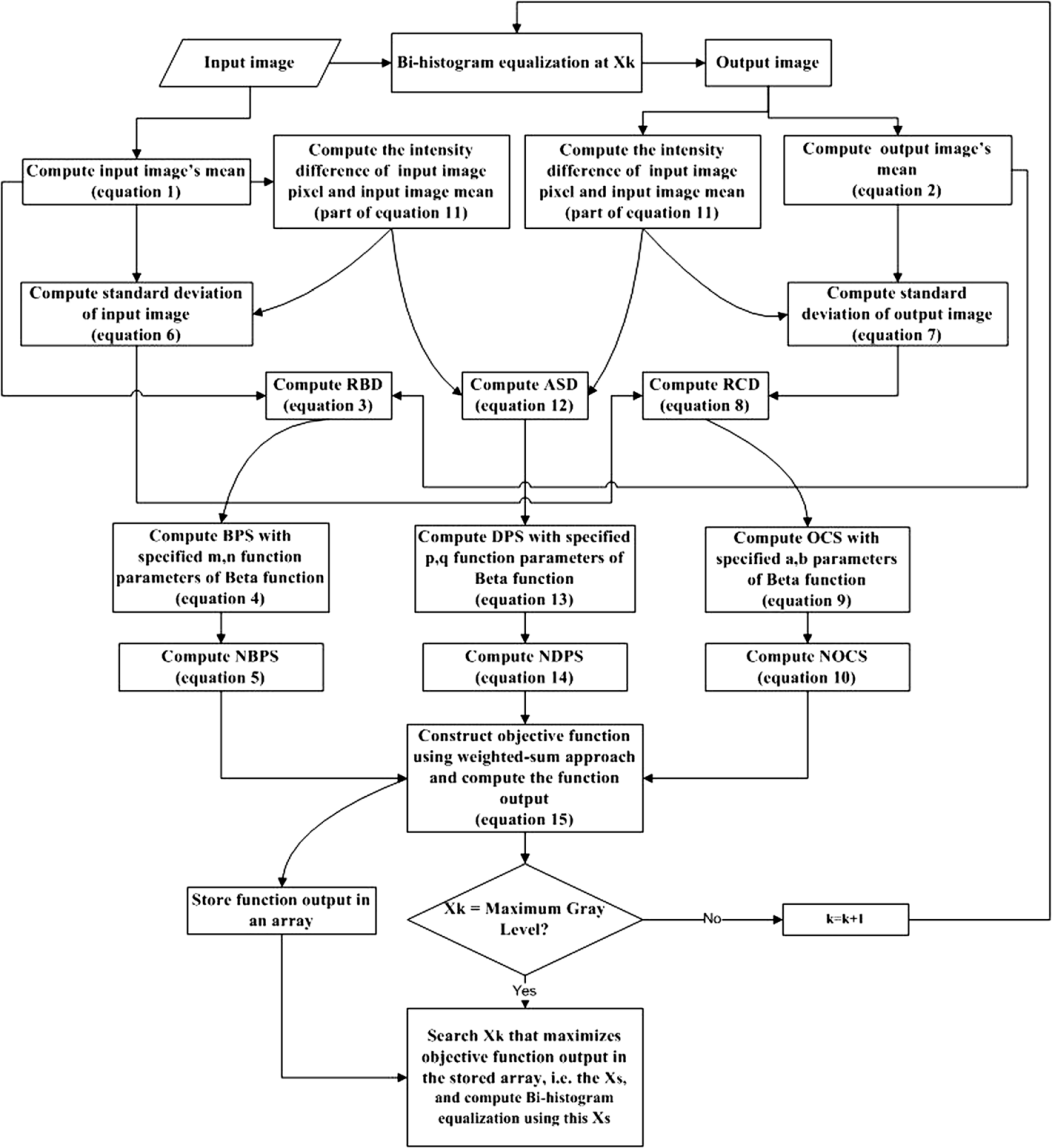
**The flowchart of the proposed algorithm.** The flowchart shows the architecture of the designed histogram equalization. This figure summarizes the description of the algorithm and facilitates understanding the flow of realizing the algorithm, with specific equation number provided in the corresponding process.

We have described so far the basic mechanism of bi-histogram equalization, the motivation for the new perspective in gauging the quality of equalized image, the potential application in bone age assessment during manual inspection, the suggested components in constructing the objective function, the construction of each component, and the combination of them into final objective function, the effect of parameters involved. To sum up everything, a block diagram showing the overall architecture of the proposed algorithm is plotted as Figure 4, with almost each block accompanied by an equation number to facilitate understanding and provide reference to enable reimplementation.

## Result and discussions

160 images from database are chosen for observer’s assessment to evaluate the effect of the improvement to the bone age assessment in terms of accuracy and time consumption.

### Observer evaluations on the effect of proposed enhancement technique on BAA

We expanded the experiments to 160 images of the adopted database to examine the effect of these human-visual-correlated Nobj to the bone age assessment (TW3 method). The adopted database implemented in testing and analyses are acquired from hand bone online database, http://www.ipilab.org/BAAweb/, which comprises of both genders in four populations which are Caucasian, African American, Hispanic, and Asian, ages ranging between 0 and 18, collected from Children’s Hospital Los Angeles (CHLA). The mentioned ossification sites refer to ulna, radius, metacarpals, I, III, V; proximal phalanges I, III, V; middle phalanges III, V; distal phalanges I, III, V; capitates, hamate, triquetral, lunate, scaphoid, trapezium, trapezoid [5].

The details of the conducted experiment are as follow:

1. Three Observers (no experience) involves in this experiments.

2. There are 3 sets of 160 hand radiographs, total of 480 images: original hand radiograph without any enhancement, denoted (set 1); general histogram equalization (GHE) denoted (set 2); proposed histogram equalization (MBOBHE) denoted (set 3).

3. Observers examined the given hand radiograph randomly from 480 images by inspecting the ossification sites and then assigning the maturity stage for each ossification site in accord to TW3 rating system (comparing the descriptions of criteria and diagrams in [5]).

4. The time taken for deducing the ossification site maturity stages is recorded.

5. Stages are converted to maturity scores according to gender and type of bones (carpal or radius, ulna and short bones (RUS)).

6. The maturity scores are totaled up and converted to bone age using conversion table in [5].

7. Each of the calculated bone age of each radiograph by the stages determined by three observers is then compared to the known chronological age of each radiograph.

8. The discrepancy (in year) between calculated bone age and chronological age are recorded for all 480 images of each observer. The averages of this discrepancy of each observer of each set of 160 images are computed and tabulated in Table 1.

9. The average time taken (in minute) to determine all the ossification sites of each radiograph of each observer of each set of 160 images are computed and tabulated in Table 2.

**Table 1:** Average discrepancy (year) between deduced bone age and chorological age

**Data Set**	**Observer 1**		**Observer 2**		**Observer 3**		**Mean**		**Total**
	**Carpal**	**RUS**	**Carpal**	**RUS**	**Carpal**	**RUS**	**Carpal**	**RUS**	
Set 1	0.66	0.43	0.69	0.58	0.54	0.49	**0.63**	**0.50**	**1.13**
Set 2	0.78	0.65	0.82	0.72	0.67	0.62	**0.76**	**0.68**	**1.44**
Set 3	0.60	0.44	0.68	0.56	0.50	0.48	**0.59**	**0.49**	**1.08**

**Table 2:** Average taken time (minute) to determine the maturity stage

**Data Set**	**Observer 1**		**Observer 2**		**Observer 3**		**Mean**		**Total**
	**Carpal**	**RUS**	**Carpal**	**RUS**	**Carpal**	**RUS**	**Carpal**	**RUS**	
Set 1	3.43	5.35	3.41	4.82	3.49	5.12	**3.44**	**5.10**	**8.54**
Set 2	3.22	5.83	3.27	4.43	3.21	5.02	**3.23**	**5.09**	**8.32**
Set 3	2.21	3.66	2.58	3.25	2.24	3.61	**2.34**	**3.51**	**5.85**

From Table 1, we found that, despite the enhancement, the accuracy of deduced bone age has no large difference; the discrepancy has only insignificant improvement (mean comparison between set1 and set2). However, it is interesting to find that general histogram equalization worsen the judgment of observers; this can be observed from the relatively higher total and mean of average discrepancy of set 2 for both carpal and RUS rating. This indicates to us that the proposed histogram technique in this observer experiment slightly improves the rating, but the improvement is not significant. More importantly, it shows that improper histogram equalization might deteriorate the final result of bone age assessment instead of improving it, due to radiographs degradation by artifacts after histogram equalization.

From Table 2, we observed that the total time taken to determine the maturity stages has improved by 31.9% (carpals), 31.2% (RUS) and 30.5% (total). This is expected since the algorithm is capable of producing resultant ossification sites that are correlating with human visual perception: the features of maturity become more distinguishable (without ‘destructive’ artifacts that impede inspection) and thus the criteria can be easily identified, leading to relatively shorter required time in determining the maturity stages.

## Conclusions

In this paper, we have proposed histogram equalization that aims to produce holistic resultant ossification sites that is capable of emphasizing the pertinent features by simultaneously considering the properties of luminance preservation, detail preservation, over-enhancement problem, and contrast enhancement. Structures have become more distinguishable to facilitate inspection for maturity stage. Experimental result has shown that the resultant image, to some extent, correlates better with human visual perception in comparison to existing methods since it considers more aspects in the attempt of avoiding artifacts. Besides, result has shown that the time taken to assess the bone age has been improved significantly while the accuracy remains the same or become slightly better than using the unprocessed radiographs. More importantly is its potential usage due to its flexibility and generality; further improvement can be done by adding in new properties in the final objective function such as the recursive separation, dynamic gray level allocation, histogram clipping using plateau limit and histogram weighting.

## Competing interests

The authors declare that they have no competing interests.

## Authors’ contributions

HYC designed the proposed algorithms for contrast enhancement of hand bones features. TTS and GHS conducted the qualitative analysis and together with HYC they finalized the interpretation. All authors read and approved the final manuscript. LKH handled the observer evaluations and the conducted experiments.
